# Individual Variation in Influenza A Virus Infection Histories and Long-Term Immune Responses in Mallards

**DOI:** 10.1371/journal.pone.0061201

**Published:** 2013-04-23

**Authors:** Conny Tolf, Neus Latorre-Margalef, Michelle Wille, Daniel Bengtsson, Gunnar Gunnarsson, Vladimir Grosbois, Dennis Hasselquist, Björn Olsen, Johan Elmberg, Jonas Waldenström

**Affiliations:** 1 Centre for Ecology and Evolution in Microbial Model Systems (EEMiS), Linnaeus University, Kalmar, Sweden; 2 Division of Natural Sciences, Kristianstad University, Kristianstad, Sweden; 3 Centre de coopération Internationale en Recherche Agronomique pour le Développement (CIRAD), Montpellier, France; 4 Department of Biology, Lund University, Lund, Sweden; 5 Department of Medical Sciences, Uppsala University, Uppsala, Sweden; University of Georgia, United States of America

## Abstract

Wild dabbling ducks (genus *Anas*) are the main reservoir for influenza A virus (IAV) in the Northern Hemisphere. Current understanding of disease dynamics and epidemiology in this virus-host system has primarily been based on population-level surveillance studies and infection experiments conducted in laboratory settings. Using a combined experimental-natural approach with wild-strain captive mallards (*Anas platyrhynchos*), we monitored individual IAV infection histories and immunological responses of 10 birds over the course of 15 months. This is the first detailed study to track natural IAV infection histories over several seasons amongst the same individuals growing from juvenile to adults. The general trends in the infection histories of the monitored birds reflected seasonal variation in prevalence at the population level. However, within the study group there were significant differences between individuals in infection frequency as well as in short and long term anti-IAV antibody response. Further observations included individual variation in the number of infecting virus subtypes, and a strong tendency for long-lasting hemagglutinin-related homosubtypic immunity. Specifically, all infections in the second autumn, except one, were of different subtypes compared to the first autumn. The variation among birds concerning these epidemiologically important traits illustrates the necessity for IAV studies to move from the level of populations to examine individuals in order to further our understanding of IAV disease and epidemiology.

## Introduction

Genuine understanding of disease dynamics requires a comprehensive knowledge of all relevant spatial and temporal variables. For diseases where migratory birds are natural hosts, it is necessary to consider migratory routes and patterns, population dynamics, as well as distinct variations among individual hosts [1]. At higher levels, samples taken at different times from different animals can be used to address questions regarding geographic distribution and prevalence of disease in a species or a population. However, individual and age-related differences in susceptibility and immune responses are important determinants for disease dynamics and host-pathogen evolution. Consequently, in order to investigate how a previous infection may affect later encounters with the same agent in terms of immunological responses, repeated measures of infection status from the same individuals over time are needed. In this context, a general problem when studying natural infections in wild animals is the difficulty of monitoring the shifting states in health and immunity of individuals over time, particularly for pathogens that do not cause overt signs of disease or acute illness [1]–[3].

Research on influenza A virus (IAV) infections in wild birds illustrate this general lack of individual-based knowledge. The majority of IAV subtypes are restricted to wild birds, particularly to dabbling ducks (genus *Anas*) [4]–[6]. However, some of the virus subtypes can also cause infections in other animal species, for example H5 and H7 that can cause severe disease in poultry [4]. In mallard, *Anas platyrhynchos*, the most studied host species of IAV, infection is most often associated with subclinical effects [7], and the bird's infection status must therefore be determined by molecular methods, or through virus propagation in fertilized eggs [8]. Disease monitoring in Eurasia and North America has provided a basic understanding of temporal and spatial variation in IAV prevalence within and amongst populations (e.g. [4], [6], [7], [9]), while the level of herd immunity in the form of anti-influenza antibodies (usually measured as anti-nucleoprotein [NP] antibodies) provides more general information on infection history of populations (e.g. [10]). For example, in the Northern Hemisphere, the annual pattern is similar across years, with high IAV prevalence levels at post-breeding aggregations and autumn migration, followed by a decrease during winter season and a low level prevalence that is maintained during spring migration and breeding [4]–[6], [11]. NP-antibody levels show similar temporal trends at the population level [10], [12]. However, understanding ducks' individual immune responses over time in the IAV study system is challenging, and relatively few studies have addressed this important aspect of IAV disease dynamics [13]–[16]. Widespread co-circulation of several different IAV subtypes, and the fact that the avian hosts are migratory and gregarious over much of the year, make studies of epidemiological processes complicated. The mobility of birds allow viruses to travel with their host along migratory flyways, providing opportunities for intrapopulation spread and subsequent IAV reassortment, which contribute to the constant emergence of novel strains [4], [5].

Individual susceptibility to infections depends on the interplay between environmental factors influencing physical condition and the costs of raising and maintaining an immunological defence against infections in relation to other life history traits [17]–[21]. Numerous factors influence both susceptibility to infection and development of immunity, including genetic variability, past infection history, physical condition, nutrition availability, abiotic conditions and the co-evolutionary history between host and pathogen [21], [22]. Wild *Anas* ducks may be infected with IAV on multiple occasions during the same autumn [7]. However, the number of infections, the duration of each singular infection and the number of virions shed during these different infection episodes are largely outside the range of population-based analyses. Studies of IAV herd immunity have illustrated that the seroprevalence (the proportion of individuals with detectable antibodies to a particular pathogen) of NP-antibodies is high among birds in autumn. Furthermore, data from ducks sampled on the wintering grounds suggest that the seroprevalence remains high, although the prevalence level of the virus itself is significantly lower during the winter [12], [21], [23]. Furthermore, despite data suggesting weak humoral responses and poor immunological memory in birds [24]–[27], laboratory studies have illustrated that mallards can produce high titres of HA-inhibiting antibodies, and that in some cases this immunity seems to be maintained for several months [13]. Such laboratory studies have also revealed individual variation in susceptibility to influenza and in immune responses [13], [15], [28]. Nevertheless, longitudinal studies of individual hosts under natural (or close to natural) settings are lacking.

This is of serious concern, as it is not at all certain that the susceptibility/resistance and virus shedding characteristic of ‘the average bird’ has the most influence on transmission and disease dynamics. Instead, for a rather benign disease such as IAV in dabbling ducks, we might expect that the most effective virus transmitters are birds that are in some respect distinct from most other individuals. This could, for example, be the fraction of ducks most susceptible to IAV infection, but it could also be the fraction mounting low immune responses against IAV (i.e., the immunologically more tolerant individuals; [29]). These factors could possibly influence both susceptibility to IAV and the duration of virus shedding. In order to learn more about susceptibility and immunity, individual-based, longitudinal studies are crucial.

To address this general knowledge gap we conducted an individual-based, long-term study. In this study, we utilized an experimental system with wild-strain mallards kept in captivity in an outdoor enclosure (i.e. sentinel ducks), in which abiotic factors, including water, were shared with wild waterfowl. Using a daily sampling regime, we constructed complete IAV infection histories for 10 birds for up to 15 consecutive months. In addition, blood samples were taken every 14 days to monitor the development and maintenance of humoral immune responses against IAV. Hence, not only do we describe long-term general trends of IAV infection in ducks, we also illustrate specific infection episodes and immune responses in individuals over time. This experimental approach allows us to conclusively illustrate changes in immune patterns and infection characteristics in the same individual as they go from immunologically naïve to a more mature state. We also show that while these patterns are generally similar among individuals, there are also some significant differences that provide useful insights into disease dynamics. Finally, we examine in detail the relationship between natural IAV infections and humoral immune responses.

## Results

### Individual IAV infection status

The sentinel ducks were sampled on 82 days in 2009 (September–December) and 238 days in 2010 (April—November). Heavy rain and snowfall made the trap and birds inaccessible 24–26 November 2010. One of the sentinel ducks (ring number 90A82120) died during the winter, thereby reducing the sample size to 9 ducks in 2010. We did not perform any postmortem investigation on the duck that died, but do not believe it was due to IAV infection as it had been IAV negative for several weeks prior to its death.

**Figure 1:**
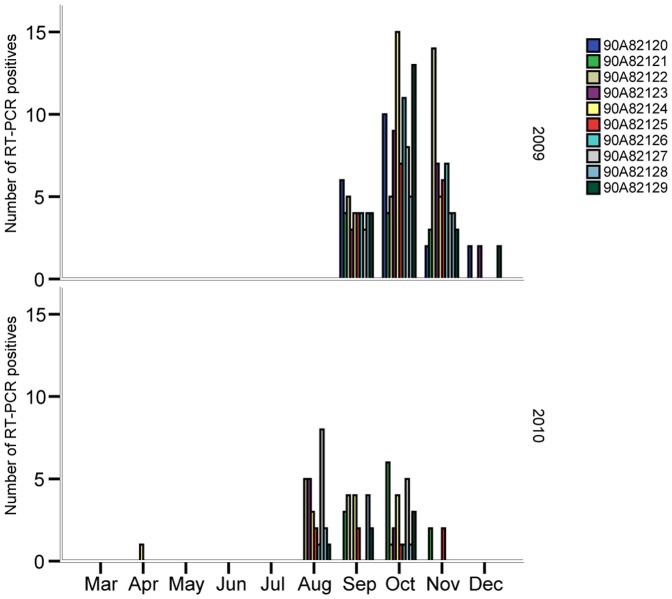
Number of influenza A virus infection (RRT-PCR positive) days per month in 2009 and 2010 given for 10 individual mallards kept under close to natural conditions in close proximity to wild mallards.

**Figure 2:**
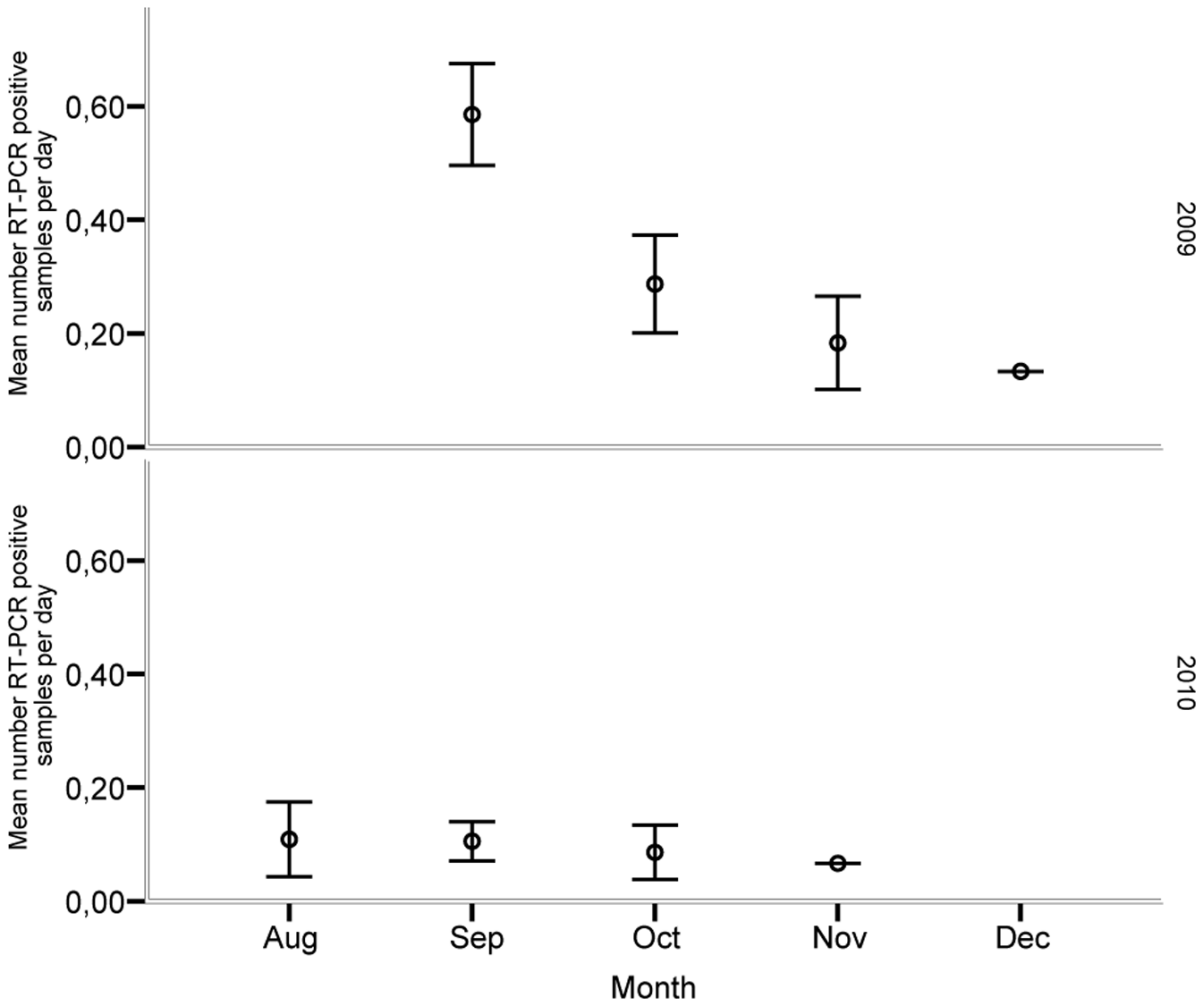
Mean number of influenza A virus (RRT-PCR) positive samples per day over the autumn months in 2009 and 2010 (corrected for sample effort). Means and 95% confidence intervals are provided, based on samples taken every day of 10 mallards kept under close to natural conditions in close proximity to wild mallards.

During the sampling period a total of 2970 samples were collected of which 226 (8.95%) were determined as IAV positive by RRT-PCR (Figure 1). On average, 19.1 infection-positive days per individual (range 11–24) were noted in 2009, while the corresponding number was 8.3 days (range 2–13) in 2010. With one exception (April 13, 2010), all detected infections occurred in the autumn, from August to December. All individuals were immunologically naïve prior to being placed in the trap, as determined by NP-ELISA and RRT-PCR. However, within the first 5 days in the trap all ducks were naturally infected with IAV. Moreover, during autumn 2009 all individuals were RRT-PCR positive on at least one occasion in September, October and November, whereas only 3 individuals were RRT-PCR-positive in December. In the following year (2010), October was the only month in which all 9 individuals were RRT-PCR positive. The only individual that was infected in the spring of 2010 was also infected on multiple occasions in September–November 2009 and in August–October 2010 (see bird 90A82124 in Figure 1). Moreover, daily monitoring of IAV infection showed that individual infection frequencies declined during the last part of both sampling seasons (Figure 2).

Virus propagation was successful for 48.2% (in 2009) and 36.6% (in 2010) of the RRT-PCR positive samples, with no difference in isolation rate depending on sample type (χ^2^ test: χ^2^<0.01, *P* = 0.998). Characterization of these viruses identified 15 different subtypes in 2009, while only 5 different variants were found in 2010 (Table 1). In the autumn of 2009, the number of distinct subtypes per bird varied from 2 to 6 (mean = 4.3). The corresponding values in 2010 varied from 0 to 4 (mean = 1.7), with no correlation between an individual's number of virus subtypes and number of infection-positive days (Spearman rank correlations: 2009: *r*_s_ = 0.185, *n* = 10, *P* = 0.609; 2010: *r*_s_ = 0.097, *n* = 9, *P* = 0.804). The dominant subtypes in 2009 were H6N2 (32 isolates from all 10 birds), H4N6 (23 isolates from 8 birds), and H11N2 (9 isolates from 5 birds; Table 1). In 2010, the five isolated subtypes were H3N8 (10 isolates from 5 birds), H3N6 (6 isolates from 2 birds), H7N7 (5 isolates from 3 birds), H11N9 (3 isolates from 2 birds and H2N2 (3 isolates from 3 birds). Interestingly, only two of the 15 hemagglutinin (HA) and neuraminidase (NA) subtype combinations found in the first season were isolated in the second season (H11N9 and H2N3). Actually, the only case of a HA-related homosubtypic infection between seasons at the individual level was in bird 90A82124, infected with a H11N2 virus in 2009 and with a H11N9 in 2010 (Table 1). At the HA Clade level, four out of the five clades detected in 2009 were also detected in 2010, but the dominating subtypes in the clades differed between the seasons, where viruses that were rare or absent in the first year dominated in the second year (Table 1). For instance, H2N3 of the H1 Clade was only isolated once in 2009 (1 out of 48 H1 Clade virus isolates in 2009), but was the only H1 Clade virus isolated in 2010 (3 out of 3 H1 Clade virus isolates in 2010).

**Table 1:** A summary of subtype information for retrieved influenza A virus isolates per individual and year.

	H1							H3				H7		H9		H11				
	H1N1	H1N2	H2N3	H5N2	H5N3	H5N9	H6N2	H3N6	H3N8	H4N3	H4N6	H7N7	H10N1	H8N4	H12N5	H11N1	H11N2	H11N9	No. Isolates^b^	No. Isolates/individual^c^
**2009**	7	1	1	1	4	2	32			1	23		1	1	3	1	9	5	4.3	92
90A82120^a^	5				1		1						1				1		5	9
90A82121						1	2							1			1		4	5
90A82122					2		4				9							2	4	17
90A82123			1	1	1		4				3							2	6	12
90A82124							2				1				2		3		4	8
90A82125							4				2								2	6
90A82126							7				3						1		3	11
90A82127	1						4				2					1		1	5	9
90A82128		1					1			1	2								4	5
90A82129	1					1	3				1				1		3		6	10
**2010**			3					6	10			5						3	1.9	27
90A82121			1									2							2	3
90A82122								2											1	2
90A82123									4										1	4
90A82124									2									1	2	3
90A82125									2			2							2	4
90A82127								4											1	4
90A82128			1						1										2	2
90A82129			1						1			1						2	4	5
**TNI** d	**7**	**1**	**4**	**1**	**4**	**2**	**32**	**6**	**10**	**1**	**23**	**5**	**1**	**1**	**3**	**1**	**9**	**8**		**119**

a Ring number of the individual bird.

b Number of isolates per individual and year.

C Number of isolates per individual.

d Total number of isolates.

Subtypes are grouped in HA Clades.

### Virus shedding

**Figure 3:**
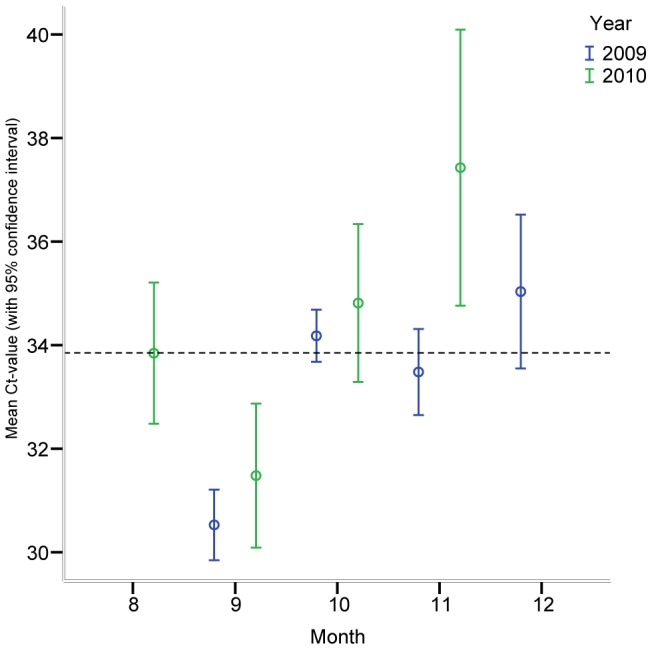
Changes in influenza A virus shedding (measured as C*_T_*-value in RRT-PCR positive faecal samples) with progress of autumn in 2009 and 2010.

Data on virus shedding were based on the C*_T_*-values from the RRT-PCR analyses, using a C*_T_* cut-off value of 40 for positivity. For each autumn season, the final ANOVA models found support for an effect of month (C*_T_*-values increasing from August to December: 2009: F = 20.07, df = 3, *P*<0.001; 2010: F = 4.80, df = 3, *P* = 0.012; Figure 3), but not of individual (2009: F = 1.24, df = 9, *P* = 0.316; 2010: F = 0.46, df = 8, *P* = 0.862). In other words, the relative intensity of virus shedding decreased in all individuals with the progress of autumn in both years.

### Immune responses

**Figure 4:**
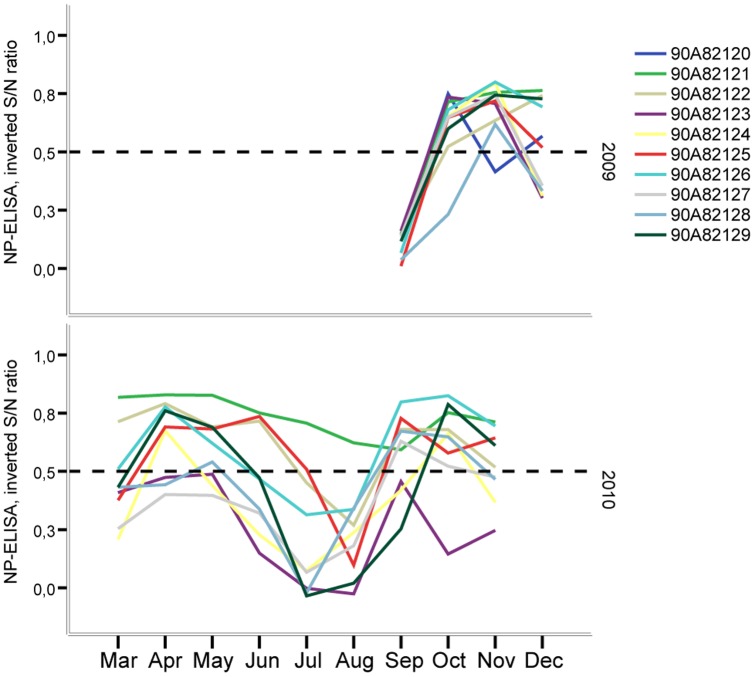
Temporal change of anti-NP-antibodies in 10 mallards kept under close to natural conditions in close proximity to wild mallards. Data are presented as the inversed monthly mean of the sample to negative control ratio for each individual. The cut-off for positivity is shown by a hatched line. Colours correspond to Figure 1.

All individuals were seronegative at the beginning of the experiment in 2009. However, the sentinel birds rapidly started to produce anti-NP antibodies after being exposed to IAV from wild ducks. Indeed, by the first bleeding interval (i.e., within 14 days after being put in the trap) all birds had seroconverted. During the first autumn (2009), 9 of 10 birds remained seropositive until the end of November, after which half of the individuals showed immune responses that were below the suggested test cut-off for presence of IAV antibodies (Figure 4). In spring (mid-March to the end of May 2010), most individuals remained seropositive, or had an anti-NP reactivity at the cut-off level. During summer (June—August), the level of anti-NP responses declined, and all but one individual were seronegative. In their second autumn, all individuals again showed increased anti-NP antibody titers, and all birds except one were seropositive throughout autumn 2010 (Figure 4). One of the ducks that showed a comparably low anti-NP response in 2009 (individual 90A82128, Figure 4) had an immune response similar to that of the other ducks in the following autumn. Conversely, the individual that showed a comparably low antibody response in 2010 (90A82123) had a strong initial antibody response in 2009, but seroreverted later in December 2009 and remained negative for the rest of the study period. The bird that was infected in the spring of 2010 (90A82124; Figure 1) showed a complex pattern of seroconversion and seroreversion during the study period. Like the other individuals, it rapidly seroconverted after infection in autumn 2009, followed by seroreversion in November. This bird was seronegative prior to the infection in April 2010, but seroconverted after the infection, as identified by the next blood collection interval, but seroreverted again later in May and remained below the threshold until August 2010 (Figure 4).

Total Ig of sentinel birds were measured and used as an indicator of general status of humoral immunity. These values showed that there was a considerable variation in Ig titers among the birds on different sampling occasions. However, despite this variation there seemed to be a weak general trend of decreasing Ig levels towards the end of each sampling season (Figure S1). It is also noticeable that the marked drop for the anti-NP-antibodies observed during summer seasons among individual birds was however not reflected by measured total Ig levels (Figure 4 and S1).

## Discussion

We found considerable individual variation among mallards in susceptibility to IAV infection and associated humoral responses. These differences were manifested despite the fact that we monitored sentinel ducks that had been raised in the same enclosure under similar conditions by a commercial breeder, that the ducks were of the same age and sex, and that they shared the same environment during the entire experiment. RRT-PCR screening of samples from individual sentinels showed that the number of days each bird was infected by IAV varied considerably in both seasons, as did the number of virus subtypes isolated during infections. On average, the number of infection days and the number of isolated subtypes were twice as high in the first autumn season, when the birds were juveniles, than in the second season, when they were adults (Tabel 1 and Figure S2). This variation is most likely linked to differences in susceptibility and resistance, rather than to differences in exposure to virus. The sentinel birds were in close proximity to each other during feeding, preening and resting, and we find it unlikely that they were not exposed to the same viral variants brought by visiting wild ducks into the adjacent compartment of the trap, or to the infections of the other sentinel ducks. Interestingly, the subtype data indicated long-lasting homosubtypic immunity related to HA subtype, where the dominating subtypes in infections in 2009 were absent in the autumn of 2010. For instance, the H4N6 and H6N2 subtypes that infected most individuals in 2009, and which have been common in the studied wild mallard population for the last ten years [11], were not found in sentinels in 2010. In fact, no reinfection with either H4 or H6 viruses was found between seasons, and the overall pattern of HA/NA subtype presence/absence was strongly influenced by year (Table 1). A formal statistical analysis of the effects of past infection histories on the likelihood of acquiring new infections with the same or different HA subtypes is not possible with the data at hand due to low statistical power and inter-dependence of data. However, a qualitative evaluation of the sentinel subtype data indicate a strong homosubtypic immunity effect, and conforms to results from experimental infections [16], [25], although these experiments have typically been run for shorter periods in a laboratory setting.

Individual differences in responses to IAV infections have been reported in other studies. Recently, Jourdain et al. [16] found that four out of six IAV naïve mallards responded with increased body temperature after experimental infection by low-pathogenic avian influenza virus, while the remaining two birds showed no such response. The same study also reported individual differences in the humoral immune response, in terms of the duration of antibody responses and in the probability of a seroconversion taking place after heterosubtypic reinfection. Similarly, in another recent study, individual variation in terms of infection frequency and duration of infection was shown for mallards of different physiological status (starved *versus* non-starved) that were infected experimentally with a low-pathogenic H5N9 IAV isolate [15]. The frequent sampling of the sentinel ducks in our study may have led to stress, which in turn may have influenced the immune status and susceptibility to infection. Further, it is possible that some individuals, although no such visual signs were observed during sampling, reacted more strongly to this stress, and that this might have contributed to differences in infection frequencies and humoral responses. However, all birds were subjected to the same sampling procedure, and daily weighing of birds showed that they followed the corresponding curves of wild-caught ducks (unpublished data), indicating that handling and sampling did not interfere with nutrition intake.

In the present study, all birds seroconverted rapidly after acquiring their first infection and the majority remained seropositive (or just below the suggested threshold) for the remainder of the first autumn. The individual anti-NP-antibody response curves showed similar topologies, with high values throughout the first autumn, a marked drop during summer, followed by a rise again in the second autumn. Despite this general trend in immunological response, we detected considerable variation in anti-IAV antibody level in different individuals on different occasions. It is interesting to note that the drop in anti-NP-antibodies is not mirrored in measurements of total Ig responses. Thus, the former is unlikely an adaptive response to balance the trade-off between immune system activation and other physiologically demanding activities (cf. [19], [30]), such as reallocation of resources from the immune system to production of eggs (the sentinel ducks laid unfertilized eggs), or production of new feathers during moult. Only one infection was found among the experimental ducks in spring, and none in summer. The same was true for wild waterfowl at the study site, where very few infections were detected in April (0 out of 366 tested samples), May (2/271), and June (2/139). The lack of detected infections in spring and the onset of new infections in the second autumn, suggest that the anti-NP-antibodies observed in spring were primarily the result of immune responses initiated the previous (first) autumn. The duration of antibody response to IAV has been a matter of debate, especially because longitudinal sampling at the individual level is very hard to achieve in wild migratory populations. In our study, anti-NP-antibody levels generally stayed high from autumn to spring, but rapidly declined in early summer, in all but one individual. Our data on the long persistence of anti-NP-antibodies are corroborated by another recent study showing that antibodies in infected mallards can persist for 6–15 months [13]. It is still unclear how antibody titers are related to the ability of an individual to clear new infections. The drop in circulating anti-NP-antibodies during summer in most individuals might result in a reduced protection against IAV. The effect of such a reduction is however not obvious, as the IAV prevalence peak in 2010 was considerably lower than that in 2009, and the fact that the subtype distributions differed substantially between the two seasons. We also found evidence for a decrease in virus shedding as autumn progressed. Taken together, these results suggest that there is some immune protection across seasons, but rather than providing an absolute protection, our data imply that there was a faster and more effective response in the second season when the birds were adult, perhaps due to immunological memory acquired during the first autumn. The subtype data indicated that homosubtypic HA immunity was frequent in the sentinel birds, but it remains to show whether this protection was specific and not related to differences in virus subtype exposure between seasons.

According to general observations of birds, the antibody peak of the first response (against a ‘new’ antigen) occurs within 12–14 days after antigen exposure, whereas the peak of a secondary (memory-based) antibody response occurs within 7–8 days (e.g., [31], [32]). Hence, the humoral defence is unlikely to be the primary protection against a gastrointestinal infection such as IAV in ducks. However, the onset of anti-NP-responses following infection in several studies conducted on mallards suggests that these antibodies are important, either serving as a mean for direct protection against the virus or by means of opsonisation in order to activate other parts of the immune system [13]. Mallards have a special set of immunoglobulin isotypes termed IgM, IgA and IgY [27]. IgM exists as a tetramer, or possibly a pentamer, and it has been shown to contribute to the early humoral response, 5–12 days post infection [33]. IgA is secreted in high concentration (5–12 mg/ml) in bile and is considered to contribute significantly to the antiviral defence in the mucosa of the gastro-intestinal tract [34]. IgY is the primary serum immunoglobulin in ducks (2–5 mg/ml) and the avian counterpart of mammalian IgG, and it replaces IgM from day 12 post infection. One particular feature of duck IgY is that it is expressed in two different forms, either as full-length IgY or as a truncated IgYΔFc variant [35], [36]. The full-length IgY variant is similar to that of other birds, including feral chicken, whereas the truncated IgYΔFc predominates at a later stage during an immune response, and because of its lack of an Fc receptor it is incapable of participating in opsonisation, complement activation and HA inhibition [37]. It has been speculated that IgYΔFc might have regulatory effect on immune reactions [27].

Population-based investigations have repeatedly found differences in IAV prevalence between age groups, with young, presumably immunologically more naïve birds, that have higher prevalence than older birds (e.g. [6], [11]). The present longitudinal study of individuals over 15 months provides a new level of resolution in this respect, including the transition from an immunologically naïve (juvenile) to an immunologically competent (adult) stage. We found that the decrease in number of IAV positive days from the first to second autumn did not correlate with a change in total Ig levels, suggesting that the observed changes in infection frequencies were not associated with a general maturation process in the humoral part of the immune system of adult birds. Our data clearly show that adult birds have an advantage in resisting IAV infections, both in terms of number of infections and in terms of duration of each infection, as compared to juvenile individuals. It should be noted that the protocol used for detecting IAV in samples collected in 2010 had approximately 10-fold higher sensitivity, and therefore the difference between the age classes is likely even greater. As all individuals became infected during their first autumn, we cannot separate age from a general immunocomptence state after a cleared infection. In this respect it would be very interesting to compare naïve and immunocompetent adults over time. It is also worth noting that specific protection was, potentially achieved by raising anti-IAV-antibodies (in this study measured as anti-NP-antibodies), drastically down-regulated during the summer months, when exposure to IAV was seemingly absent. No such down-regulation was seen in total Ig level, again suggesting a non-negligible role of the humoral response against IAV infection.

In summary, we present long-term infection and immune profiles for mallards and validate population level infection data. Further, we illustrate that higher prevalence estimates for juvenile birds is not a sampling artefact, but rather a result of the immune system being primed for a more efficient immune response as an adult and likely also having a long-lasting homosubtypic immunity component, where reinfection with similar HA subtypes tended to be rare between seasons. There is a strong relationship between infection and immune response, and we illustrate complex patterns of seroconversion and seroreversion in mallards across 15 months. We propose that variation in susceptibility, shedding times and immune response at the individual level translates into factors for disease dynamics at the population level, and indeed, we found significant individual variation both in the number of infections and in the general and subtype specific immunological responses to these infections amongst naturally infected mallards. Such detailed knowledge about the relationship between infection and immunological response is crucial to fully understand the spatial and temporal dynamics of IAV epidemiology and should be incorporated in future IAV surveillance and modeling.

## Materials and Methods

### Ethics statement

Ethical approval for trapping, sampling, and keeping birds was obtained from the Swedish Animal Research Ethics Board (“Linköpings djurförsöksetiska nämnd”, reference number 46-09).

### Study site

The study was conducted at Ottenby, a major stopover site for waterfowl on the southern tip of Öland, an island in the southern Baltic (56°13′N 16°27′E). Ottenby is located in the centre of the northwest European waterfowl flyway, which extends from northwestern Russia to France and adjacent countries [38]. Mallards and other waterfowl have been captured at Ottenby Bird Observatory since 1962 in a stationary baited duck trap situated on the shore of a brackish lagoon, partly in water and partly on land. To attract wild waterfowl, grain is provided at the entrances and inside the trap, and there is a fenced (sentinel duck) compartment permanently hosting wild-strain mallards originating from a commercial farm. Permeable walls, made of nylon mesh, enable water and natural food items such as seeds and invertebrates to freely move between the sentinel duck compartment and the surrounding environment. The trap is visited by a large number of wild ducks, particularly mallards, and daily catches can exceed a hundred ducks during peak migration in late autumn. The sentinel ducks are only separated from the wild ducks by the nylon mesh, and viruses could be transmitted via water, splashes and droplets.

### Sentinel animals and sampling scheme

We used 10 juvenile wild-strain domestic mallard females as experimental ducks. When the experiment started (24 September 2009) these birds were approximately 4 months old. They were kept in the sentinel duck compartment during autumn 2009 (24 September to 15 December), and from spring until autumn in 2010 (4 April to 30 November). During winter and early spring the birds were kept indoors in a barn due to unfavourable ice conditions at the trap and were therefore not sampled during this period. The farm had no other poultry, and thereby the risk of exposure to IAV during winter was very low. In the trap, ducks were provided with grain (wheat, rye, barley), but were also able to feed on naturally occurring food items present in the trap (e.g. seeds, invertebrates, live plant parts). Every day throughout the study, the sentinel ducks were sampled by cloacal swabbing or from fresh fecal deposits in single-use cardboard boxes. In order to avoid excessive stress for the birds during sampling as well as keeping the daily sampling effort to a minimum, no oropharyngeal samples were taken. Samples were preserved in 1 ml virus transport medium (Hank's balanced salt solution containing 0.5% lactalbumin, 10% glycerol, 200 U/mL penicillin, 200 µg/mL streptomycin, 100 U/mL polymyxin B sulfate, 250 µg/mL gentamicin, and 50 U/mL nystatin Sigma). All wild mallards caught in the trap were similarly sampled as part of an ongoing surveillance scheme.

### IAV detection, isolation and characterization

In brief, samples were thawed on ice, thoroughly vortexed, and 100 µl per sample was taken for RNA extraction. For samples collected in 2009, RNA was isolated using the M48 Biorobot with the MagAttract Viral RNA M48 extraction kit (Qiagen), while samples from 2010 were extracted with the MagNA Pure 96 robot and the Viral NA Large Volume Kit (Roche) according to the manufacturers' specifications. In subsequent real-time reverse transcriptase PCR (RRT-PCR), 2 µl of RNA was analysed using a One-step RT-PCR kit (Qiagen) together with primers and a TaqMan probe directed to the viral matrix segment [39] in a Light Cycler 1.5 (Roche), and later in a StepOnePlus (Applied Biosystems) real-time PCR system. Default settings were used to determine cycle threshold (C*_T_*) values during DNA-synthesis. RRT-PCR-positive samples were propagated in specific pathogen-free (SPF) embryonated hens' eggs using standard methods [8], [40]. The HA subtype of virus isolates was characterized using HA inhibition (HI) and/or PCR followed by sequencing, and the NA subtype was characterized by PCR and sequencing [8]. Sequenced segments were deposited in GeneBank with the accession numbers JX565989–JX566080, JX566173–JX566264.

### Virus shedding

The C*_T_*-value from the RRT-PCR screening of the viral matrix gene was used as an indirect measure of the number of virus particles present in a sample. As previously described, different RNA isolation protocols and RRT-PCR machines were used for the 2009 and the 2010 samples; hence the resulting C*_T_*-values are not directly comparable between seasons. Extensive testing showed that the equipment used in 2010 was approximately 10 times more sensitive, within a range of C*_T_*-values from 20–37, in control experiments where IAV was serially diluted 10–10^7^ times. Due to these differences we performed separate analyses for each year. The C*_T_*-values were normally distributed (Kolmogorov-Smirnov, *P*>0.05) and did not differ depending on sample type (independent sample t-test t = 0.247, d.f. = 187, *P* = 0.805; test based on the 2009 subset). A set of general linear models (GLMs) was run to test for a response in the C*_T_* variable by season and individual. In these models, C*_T_*-value was used as the dependent variable with date (defined as the number of days from 1 August) as a covariate and individual fitted as a random factor.

### Development of immunity

Blood samples were taken before the start of the experiment and then about every 14 days during the field seasons. Approximately 0.8 ml of blood was collected from the tarsal or brachial vein. Blood was transferred to MiniCollect ZSerum Seperator tubes (Greiner Bio-One GmbH, Austria) and later (from 30 min to 4 h) spun at ∼3000× g for 10 minutes to separate serum from blood cells. The resulting serum samples were stored at −20°C. All samples were checked for IAV antibodies using a competitive ELISA designed to detect avian anti-nucleoprotein (NP) antibodies (IDEXX FlockCheck*, Avian Influenza Virus Antibody Test Kit, ELISA, MultiS-Screen, IDEXX Laboratories Europe, Hoofddorp, The Netherlands). The results were interpreted following the manufacturer's instructions by determining the serum sample to negative control ratio (S/N), where ratios <0.50 were considered as positive. For visualization, we used the inverted S/N ratio in all figures.

We measured the total level of immunoglobulins (Ig) using a standard ELISA protocol designed for use on wild birds [31], [41], but with slight modification. Briefly, ELISA plates were coated with 100 ml of goat-anti-bird IgG unlabelled (Novus Biologicals, Littleton, CO) diluted 1∶2000 in carbonate buffer (0.15 M, pH 9.6). Plates were incubated overnight at 4°C and then blocked for 2 h at room temperature with 200 ml of 3% powdered milk diluted in PBS-Tween 20. The serum samples were diluted 1∶300 in diluent (1% powdered milk, PBS-Tween 20). After washing, diluted serum samples (plus standard samples and blanks) were added to the plate in duplicates. The plates were incubated a second time overnight at 4°C. On the third day, after a first wash we added 100 ml of goat-anti-bird IgG labelled with horseradish peroxidase (Novus Biologicals, Cat. No. NB 7228) that was diluted 1∶3000 in 1% powdered milk, PBS-Tween 20, and the plates were then incubated for 30 minutes at 37°C. The plates were then washed and 100 ml of peroxidase substrate (2,2-azino-bis-3-ethylbenzthiazoline-6-sulfonic acid, ABTS; Sigma cat. A1888) and peroxide were added. The plates were read on an ELISA-reader at 30-s intervals for 14 min using a 405-nanometer wavelength filter. All antibody concentrations are reported as the slope of the substrate conversion (in milli-optical densities; mOD) over time (mOD/min). We calculated the mean of the duplicate values for each sample to obtain an antibody titer value. The mean value of the blanks was subtracted from the measured antibody titer to account for non-specific binding. On each plate, we included a dilution series of a standard sample that covered the range of antibody titers for the mallards. We used the differences between the standard curves to account for between-plate variation.

## Supporting Information

Figure S1 Influenza A virus detected by RRT-PCR in individual sentinel ducks in 2009 and 2010.
(EPS)

Figure S2 Temporal change of total Ig levels in 10 mallards kept under close to natural conditions in close proximity to wild mallards. Colours correspond to those used in Figure 1.
(TIF)

## References

[1] Altizer S, Bartel R, Han BA (2011) Animal migration and infectious disease risk. Science 331: 296–302. 21252339 10.1126/science.1194694

[2] Wobeser GA (2006) Essentials of disease in wild animals. Ames, Iowa: Blackwell Publishing.

[3] Fuller T, Bensch S, Muller I, Novembre J, Perez-Tris J, et al (2012) The ecology of emerging infectious diseases in migratory birds: An assessment of the role of climate change and priorities for future research. Ecohealth 9: 80–88 doi:10.1007/S10393-012-0750-1. 22366978 10.1007/s10393-012-0750-1

[4] Olsen B, Munster VJ, Wallensten A, Waldenström J, Osterhaus AD, et al (2006) Global patterns of influenza A virus in wild birds. Science 312: 384–388. 16627734 10.1126/science.1122438

[5] Webster RG, Bean WJ, Gorman OT, Chambers TM, Kawaoka Y (1992) Evolution and ecology of influenza A viruses. Microbiol Rev 56: 152–179. 1579108 10.1128/mr.56.1.152-179.1992 PMC372859

[6] Munster VJ, Baas C, Lexmond P, Waldenström J, Wallensten A, et al (2007) Spatial, temporal, and species variation in prevalence of influenza A viruses in wild migratory birds. PLOS Pathogens 3: e61 doi:10.1371/journal.ppat.0030061. 17500589 10.1371/journal.ppat.0030061 PMC1876497

[7] Latorre-Margalef N, Gunnarsson G, Munster VJ, Fouchier RA, Osterhaus AD, et al (2009) Effects of influenza A virus infection on migrating mallard ducks. Proc Biol Sci 276: 1029–1036. 19129127 10.1098/rspb.2008.1501 PMC2679067

[8] Munster V, Baas C, Lexmond P, Bestebroer TM, Guldemeester J, et al (2009) Practical considerations for high-throughput influenza A virus surveillance studies of wild birds by use of molecular diagnostic tests. Journal of Clinical Microbiology 47: 666–673. 19109483 10.1128/JCM.01625-08 PMC2650931

[9] Wilcox BR, Knutsen GA, Berdeen J, Goekjian V, Poulson R, et al (2011) Influenza A viruses in ducks in northwestern minnesota: Fine scale spatial and temporal variation in prevalence and subtype diversity. Plos One 6: e24010 doi:10.1371/journal.pone.0024010. 21931636 10.1371/journal.pone.0024010 PMC3172203

[10] De Marco MA, Campitelli L, Foni E, Raffini E, Barigazzi G, et al (2004) Influenza surveillance in birds in Italian wetlands (1992–1998): Is there a host restricted circulation of influenza viruses in sympatric ducks and coots? Vet Microbiol 98: 197–208. 15036528 10.1016/j.vetmic.2003.10.018

[11] Wallensten A, Munster VJ, Latorre-Margalef N, Brytting M, Elmberg J, et al (2007) Surveillance of influenza A virus in migratory waterfowl in northern Europe. Emerg Infect Dis 13: 404–411. 17552093 10.3201/eid1303.061130 PMC2725893

[12] Arenas A, Carranza J, Perea A, Miranda A, Maldonado A, et al (1990) Type A influenza viruses in birds in southern Spain: Serological survey by enzyme-linked immunosorbent assay and haemagglutination inhibition tests. Avian Pathol 19: 539–546. 18679964 10.1080/03079459008418706

[13] Fereidouni SR, Grund C, Hauslaigner R, Lange E, Wilking H, et al (2010) Dynamics of specific antibody responses induced in mallards after infection by or immunization with low pathogenicity avian influenza viruses. Avian Dis 54: 79–85. 20408403 10.1637/9005-073109-Reg.1

[14] Globig A, Fereidouni SR, Harder TC, Grund C, Beer M, et al (2012) Consecutive natural influenza A virus infections in sentinel mallards in the evident absence of subtype-specific hemagglutination inhibiting antibodies. Transbound Emerg Dis doi:10.1111/j.1865-1682.2012.01357.x. 10.1111/j.1865-1682.2012.01357.x 22816511

[15] Arsnoe DM, Ip HS, Owen JC (2011) Influence of body condition on influenza A virus infection in mallard ducks: Experimental infection data. Plos One 6: e22633 doi:10.1371/journal.pone.0022633. 21857940 10.1371/journal.pone.0022633 PMC3156705

[16] Jourdain E, Gunnarsson G, Wahlgren J, Latorre-Margalef N, Bröjer C, et al (2010) Influenza virus in a natural host, the mallard: Experimental infection data. Plos One 5: e8935 doi:10.1371/journal.pone.0008935. 20126617 10.1371/journal.pone.0008935 PMC2812492

[17] Sheldon BC, Verhulst S (1996) Ecological immunology: Costly parasite defences and trade-offs in evolutionary ecology. TREE 11: 317–321. 21237861 10.1016/0169-5347(96)10039-2

[18] Verhulst S, Riedstra B, Wiersma P (2005) Brood size and immunity costs in zebra finches *Taeniopygia guttata* . J Avian Biol 36: 22–30.

[19] Hasselquist D, Nilsson JÅ (2012) Physiological mechanisms mediating costs of immune responses: What can we learn from studies of birds? Anim Behav 83: 1303–1312.

[20] Lochmiller RL, Deerenberg C (2000) Trade-offs in evolutionary immunology: Just what is the cost of immunity? Oikos 88: 87–98.

[21] Hoye BJ, Munster VJ, Nishiura H, Fouchier RAM, Madsen J, et al (2011) Reconstructing an annual cycle of interaction: Natural infection and antibody dynamics to avian influenza along a migratory flyway. Oikos 120: 748–755.

[22] Demas G, Nelson R (2012) Ecoimmunolgy. Oxford: Oxford University Press. 576 p.

[23] De Marco MA, Foni GE, Campitelli L, Raffini E, Di TL, et al (2003) Circulation of influenza viruses in wild waterfowl wintering in Italy during the 1993–99 period: Evidence of virus shedding and seroconversion in wild ducks. Avian Dis 47: 861–866. 14575078 10.1637/0005-2086-47.s3.861

[24] Suarez DL, Schultz-Cherry S (2000) Immunology of avian influenza virus: A review. Dev Comp Immunol 24: 269–283. 10717293 10.1016/s0145-305x(99)00078-6

[25] Kida H, Yanagawa R, Matsuoka Y (1980) Duck influenza lacking evidence of disease signs and immune response. Infect Immun 30: 547–553. 7439994 10.1128/iai.30.2.547-553.1980 PMC551346

[26] Capua I, Mutinelli F, Pozza MD, Donatelli I, Puzelli S, et al (2002) The 1999–2000 avian influenza (H7N1) epidemic in Italy: Veterinary and human health implications. Acta Trop 83: 7–11. 12062787 10.1016/s0001-706x(02)00057-8

[27] Magor KE (2011) Immunoglobulin genetics and antibody responses to influenza in ducks. Dev Comp Immunol 35: 1008–1017. 21377488 10.1016/j.dci.2011.02.011

[28] Costa TP, Brown JD, Howerth EW, Stallknecht DE (2010) Effect of a prior exposure to a low pathogenic avian influenza virus in the outcome of a heterosubtypic low pathogenic avian influenza infection in mallards (*Anas platyrhynchos*). Avian Dis 54: 1286–1291. 21313851 10.1637/9480-072210-Reg.1 PMC11337149

[29] Råberg L, Graham AL, Read AF (2009) Decomposing health: Tolerance and resistance to parasites in animals. Philos Trans R Soc Lond B Biol Sci 364: 37–49. 18926971 10.1098/rstb.2008.0184 PMC2666700

[30] Råberg L, Grahn M, Hasselquist D, Svensson E (1998) On the adaptive significance of stress-induced immunosuppression. Proc Biol Sci 265: 1637–1641. 9753786 10.1098/rspb.1998.0482 PMC1689346

[31] Hasselquist D, Marsh JA, Sherman PW, Wingfield JC (1999) Is avian humoral immunocompetence suppressed by testosterone? Behav Ecol Socibiol 45: 167–175.

[32] Owen-Ashley NT, Hasselquist D, Wingfield JC (2004) Androgens and the immunocompetence handicap hypothesis: Unraveling direct and indirect pathways of immunosuppression in song sparrows. Am Nat 164: 490–505. 15459880 10.1086/423714

[33] Higgins DA, Shortridge KF, Ng PL (1987) Bile immunoglobulin of the duck (*Anas platyrhynchos*). II. Antibody response in influenza A virus infections. Immunology 62: 499–504. 3451744 PMC1454141

[34] Magor KE, Warr GW, Bando Y, Middleton DL, Higgins DA (1998) Secretory immune system of the duck (*Anas platyrhynchos*). Identification and expression of the genes encoding IgA and IgM heavy chains. European Journal of Immunology 28: 1063–1068. 9541602 10.1002/(SICI)1521-4141(199803)28:03<1063::AID-IMMU1063>3.0.CO;2-O

[35] Magor KE, Higgins DA, Middleton DL, Warr GW (1994) One gene encodes the heavy-chains for three different forms of IgY in the duck. Journal of Immunology 153: 5549–5555. 7989756

[36] Magor KE, Warr GW, Middleton D, Wilson MR, Higgins DA (1992) Structural relationship between the two IgY of the duck, *Anas platyrhynchos* - molecular genetic evidence. Journal of Immunology 149: 2627–2633. 1401901

[37] Warr GW, Magor KE, Higgins DA (1995) Igy - clues to the origins of modern antibodies. Immunology Today 16: 392–398. 7546196 10.1016/0167-5699(95)80008-5

[38] Guillemain M, Sadoul N, Simon GR (2005) European flyway permeability and abmigration in teal *Anas crecca*, an analysis based on ringing recoveries. Ibis 147: 688–696.

[39] Spackman E, Senne DA, Myers TJ, Bulaga LL, Garber LP, et al (2002) Development of a real-time reverse transcriptase PCR assay for type A influenza virus and the avian H5 and H7 hemagglutinin subtypes. Journal of Clinical Microbiology 40: 3256–3260. 12202562 10.1128/JCM.40.9.3256-3260.2002 PMC130722

[40] Ellström P, Latorre-Margalef N, Griekspoor P, Waldenström J, Olofsson J, et al (2008) Sampling for low-pathogenic avian influenza A virus in wild mallard ducks: Oropharyngeal versus cloacal swabbing. Vaccine 26: 4414–4416. 18586061 10.1016/j.vaccine.2008.06.027

[41] Hanssen SA, Hasselquist D, Folstad I, Erikstad KE (2004) Costs of immunity: Immune responsiveness reduces survival in a vertebrate. Proc Biol Sci 271: 925–930. 15255047 10.1098/rspb.2004.2678 PMC1691677

