# Supplementary figures and images for: Individual Variation in Influenza A Virus Infection Histories and Long-Term Immune Responses in Mallards
Source: PLoS One. 2013 Apr 23;8(4):e61201. doi: 10.1371/journal.pone.0061201 (PMC3634042; doi:10.1371/journal.pone.0061201)

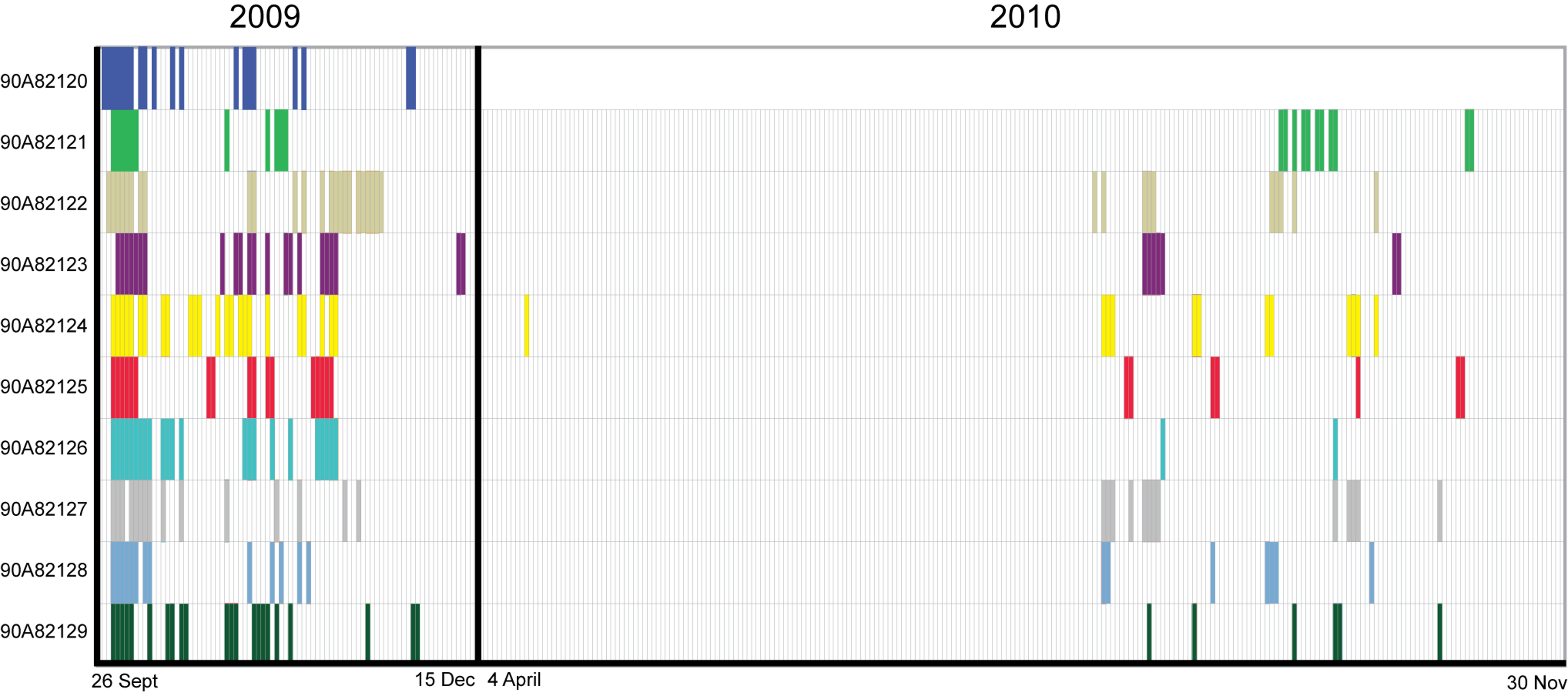

Supplement: Figure S2 — Temporal change of total Ig levels in 10 mallards kept under close to natural conditions in close proximity to wild mallards. Colours correspond to those used in Figure 1. (TIF) [file pone.0061201.s002.tif]
